# Effects of Six-Week Resistance Training with or without Vibration on Metabolic Markers of Bone Metabolism

**DOI:** 10.3390/ijerph18189860

**Published:** 2021-09-18

**Authors:** Patrick Lau, Åsa Beijer, André Rosenberger, Eckhard Schoenau, Christoph Stephan Clemen, Jochen Zange, Jörn Rittweger

**Affiliations:** 1 German Aerospace Center (DLR), Institute of Aerospace Medicine, 51147 Cologne, Germany; abeijer@gmail.com (Å.B.); Andre.Rosenberger@gmx.net (A.R.); christoph.clemen@dlr.de (C.S.C.); jochen.zange@dlr.de (J.Z.); joern.rittweger@dlr.de (J.R.); 2 Institute of Training Science and Sports Informatics, German Sport University Cologne, 50933 Cologne, Germany; 3 Department of Pediatrics and Adolescent Medicine, University Hospital of Cologne, 50937 Cologne, Germany; eckhard.schoenau@uk-koeln.de; 4 Medical Faculty, University Cologne, 50931 Cologne, Germany

**Keywords:** strength training, bone markers, sclerostin, CTX, P1NP

## Abstract

Acute and protracted effects of resistive exercise (RE) and resistive exercise with whole-body vibration (RVE) on metabolic markers of bone metabolism were investigated. Twenty-six men participated in a randomized training program including RE (*n* = 13; age = 23.4 ± 1.4 years) or RVE (*n* = 13; age = 24.3 ± 3.3 years). During the first session, acute C-terminal telopeptide of type I collagen (CTX) responses decreased by 12.9% (standard deviation, SD 13.7%) after 2 min, followed by a 15.5% (SD 36.0%) increase at 75 min after exercise (both *p* < 0.001). Procollagen type I amino terminal propeptide (P1NP) increased by 12.9% (SD 9.1%) at 2 min (*p* < 0.001) but no change occurred at 75 min. Sclerostin showed prolonged responses from 2 to 75 min post-exercise in the first session (*p* < 0.001). Acute responses at the first session were comparable between groups for CTX and P1NP, acute sclerostin responses were substantially greater in RE than in RVE (*p* = 0.003). No significant differences were noted in the resting baseline levels of CTX, P1NP, or sclerostin from the beginning to the end of the six-week progressive training. The present study therefore did not demonstrate any sizeable enhancement of bone turnover that could match the effects that have been repeatably made in response to countermeasure exercise during bed rest.

## 1. Introduction

**Figure 1:**
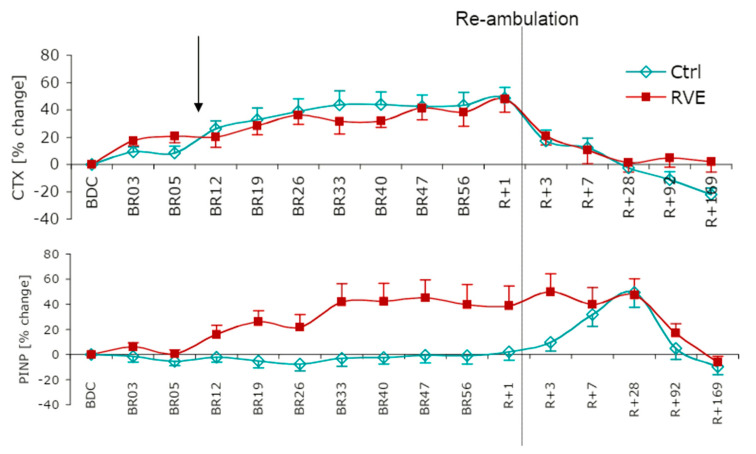
Changes in markers of bone formation and resorption during and after bed rest (8 weeks of strict bed rest; Berlin Bed-Rest Study) in each subject group. Cross-linked C-terminal telopeptide of type I collagen (CTX; bone resorption), procollagen type I amino terminal propeptide (P1NP; bone formation). The error bar represents the standard error of the mean percentage of the difference from baseline (BDC-2) values. Ctrl, control group; RVE, resistive vibration exercise group [8].

Regular physical exercise positively affects bone mass, and physical inactivity induces bone losses. Mechanical stimuli, especially strains, are thought to be pivotal in the adaptive processes in bones [1]. Consequently, mechanical loading is a potential method for increasing bone mass and improving bone strength. The anabolic effect of physical exercise on the osseous tissues is primarily related to mechanical effects, and multiple factors may interfere with the osteogenic response [2]. Physical exercise has also been found to constitute an effective countermeasure against bone loss in experimental bed rest [3,4,5], which is a ground-based analog model of spaceflight [6], with a well-established bone-catabolic response [7]. However, different than one might think, the preventive effect of resistive vibration exercise (RVE) for bone is not mediated by an anti-catabolic mechanism, but rather by a bone-anabolic effect (Figure 1) which offsets the seemingly invariant bone-catabolic response to bed rest [8].

This led us to wonder how bone formation and resorption are affected when a resistive vibration exercise protocol, which is effective during experimental bed rest [4], is performed under ambulatory conditions. In this context, there exist sensitive and specific serum markers of bone metabolism that are especially useful for investigating the acute effects of exercise on bone remodeling [9]. Specifically, the cross-linked C-terminal telopeptide of type I collagen (CTX), which is a split product from bone-specific protein released to the blood serum and therefore a marker of collagen breakdown, accurately reflects the level of bone resorption [10]. Pro-collagen type I amino terminal propeptide (P1NP) is released in the serum during the polymerization of bone-specific collagen. Hence, the serum levels of P1NP reflect bone formation [11]. The response is orchestrated by osteocytes upstream of osteoblast and osteoclast activity [12]. Sclerostin is one of the key osteokines [13]. It constitutes an osteocyte-specific secreted protein and appears to be a potent inhibitor of the canonical Wnt/β-catenin pathway, a well-established factor in bone mass control [14]. Sclerostin has been reported to be an adequate signal of bone turnover that is involved in bone remodeling and may reflect the activity of osteocytes [15]. The expression by osteocytes and the resulting serum levels of sclerostin are increased by mechanical forces acting on bones and by hormones known to affect bone metabolism, such as parathyroid hormone, calcitonin, and glucocorticoids [16,17]. There is currently a growing interest in the discovery of effective exercise interventions to improve bone structure and health in multiple populations [18]. Thus, basic approaches are important, as bone adapts to mechanical loading signals [19,20,21]. Whole-body vibration has been suggested as an exercise modality that is particularly suitable for preservation of bone strength [22,23]. The aim of the present study was to investigate the acute osteogenic response to exercise training, and whether or not these responses habituate after repeated exposure. We therefore defined a six-week strength intervention study. It was anticipated that substantial habituation of acute responses, if it exists, would also be observable, even if a six-week training program is somewhat shorter than the typical eight-week duration for resistive exercise studies. As a general guideline, exercise usually is conducted and recommended on a regular basis (e.g., 2–3 times a week) for a longer period of time. We took great care to run all training sessions with supervision, and to achieve a high completion rate. We hypothesized that bone turn-over would be enhanced at the end of the six-week intervention and that this effect would be more pronounced with RVE than with resistive exercise (RE) alone. Moreover, we were interested in monitoring acute bone marker responses to the first and last exercise session within the six-week training program.

## 2. Materials and Methods

The Effects of Vibration Exercise Study was conducted as a stratified, randomized, two-group, parallel design. A detailed description of the exercises and study design has been published elsewhere [24]. Notably, all described effects were noticed within the observational period of 6-weeks. Therefore, previously published results from the same study have demonstrated gains in leg muscle size and function [25], as well as adaptive responses in the supplying arteries and microcirculation [26,27,28], thus demonstrating that both interventions took effects, albeit somewhat differentially. Within a given subject, the first and last training session was always scheduled at the same daily time to negate circadian effects on bone markers. Dietary food intake was controlled on the days of the first and last training session. Subjects ate a standardized breakfast consisting of two wheat bread rolls with butter and jam 2 h before training. During the training intervention, subjects were asked to abstain from food 2 h before every training session and to drink a protein energy drink (Fresubin protein energy drink, Fresenius Kabi, Bad Homburg vor der Höhe, Germany) 1 h before training started.

### 2.1. Study Participants

In total, 26 healthy and physically active male subjects were included in the study after obtaining written informed consent. The study was officially conducted in compliance with the Declaration of Helsinki, following approval by the Ethics Committee of the Northern Rhine Medical Association (Ärztekammer Nordrhein) in Düsseldorf (application No. 2010-174). All subjects were healthy, physically active subjects (exercised 2–3 times per week). The exclusion criteria included participation in any competitive sports, participation in strength training during the past 6 months, smoking, diabetes, and currently taking any medications. Notably, there was no difference between the two groups (Table 1). The acute responses of bone markers were measured 2–75 min after exercise. To test whether the training changed these acute responses before and after the first and last training session, examinations were repeated after 6 weeks of either RE or RVE training. It was therefore anticipated that substantial habituation of acute responses would also be observable after such a training interval.

### 2.2. Exercise Protocols

All subjects trained 2–3 times per week for 6 weeks. In detail, 10 subjects in the RE group completed all 16 training sessions, whereas the remaining 3 subjects missed 1 training session. The mean age of the subjects in the RE group was 23.4 ± 1.4 years; mean body weight 75.0 ± 4.7 kg. In the RVE group, 4 subjects completed all 16 training sessions, and 9 subjects missed a single training session. Thus, 413 out of 416 training sessions were successfully completed, yielding a completion rate >99%. The RE included training with weights on a guided barbell (PTS Dual-Action Smith, Hoist, CA, USA). The individual training load was set to 80% of their one-repetition maximum (1-RM). Squat performance was measured before the initial training session, which was determined using the method described by Baechle et al. [29]. Each session consisted of three sets of eight squats (with 2-s eccentric and 2-s concentric phases) and 12 heel raises (with 1-s eccentric and 1-s concentric phases), separated by a 1-min break. To adapt the loading for the following training session accordingly, the last set was performed with the maximum possible number of repetitions for squats and heel raises. A metronome was used to guide the rhythm of movement. Each exercise session consisted of a warm-up of two sets of 10 squats and 15 heel raises with an unloaded barbell (15 kg). Subjects allocated to the RVE group mean age, 24.3 ± 3.3 years; mean body weight 74.7 ± 6.9 kg performed the same exercise protocol as the RE group but with the addition of the side-alternating whole-body vibration platform (Galileo Fitness, Novotec Medical GmbH, Pforzheim, Germany) with a 6–8 mm peak-to-peak displacement and frequencies between 20 and 40 Hz. The vibration frequency was gradually increased by 5 Hz/week to reach a maximum of 40 Hz during the last 2 weeks of training.

### 2.3. Biochemical Analyses

We collected fasted baseline blood samples and measured the concentrations of bone metabolism markers initially and immediately after the final session of the 6-week training intervention. Blood was collected from the cephalic vein using a short catheter 1 h prior to exercise as well as at +2 min, +5 min, +15 min, +35 min, and +75 min after exercise. The blood samples were then deposited into serum monovettes (Sarstedt, Nürmbrecht, Germany), allowed to clot for 10 min, centrifuged at 3000 rpm (1500× *g*) at 4 °C (Heraeus Multifuge 1S-R, Thermo Scientific, Waltham, MA, USA), distributed into small collection tubes, and immediately frozen at −80 °C until the time of analysis. All analyzed biomarkers and regulators of bone metabolism were analyzed by commercially available immunoassays. Bone resorption was assessed by sCTX-I (Immundiagnostic Systems, UK; REF AC-02F1), and bone formation was elevated by intact N-terminal P1NP (RIA Orion Diagnostica, Finland; REF 67034). Serum sclerostin was measured using a sclerostin enzyme-linked immunosorbent assay (Quidel, San Diego, CA, USA; REF TE1023HS) according to the manufacturer’s instructions. To avoid inter-assay variation within one subject, samples from each subject were analyzed in one batch. Inter- and intra-assay variations, respectively, were as follows: CTX-I: 6.8%, 5.9%; P1NP: 2.1%, 2.3%; and sclerostin both 2.1%.

### 2.4. Statistical Analysis

We performed the statistical analyses using the R-environment program version 3.5.1 for the 64-bit Windows platform (www.r-project.org; accessed on 29 August 2018). Linear mixed effect (LME) models were constructed using the R-function “lme”. To address the primary question, namely whether a 6-week training program of RE or RVE would differentially affect metabolic markers of bone metabolism, we used only resting values (i.e., before the acute exercise training) of each session, and we applied LME models with subject ID as a random factor and group (RVE vs. RE) and session (either first training session or last) as fixed factors, as well as a group × session interaction term. Significant analysis of variance effects were followed up by a priori treatment contrast with RE and the first session as reference. To address the second question, namely whether the 6-week intervention would affect the acute bone marker responses to exercise, we computed the percentage changes from resting baseline for each session. With these percentage changes, we constructed LME models that also included time (resting baseline levels and +2 min, +5 min, +15 min, +35 min, and +75 min after exercise) into the model, allowing all possible two-way interactions and also the three-way interaction term of group × session × time. Where data transformation was required, as judged by residual plots and by qq-plots, we used an optimized box-cox transformation [30]. The simplification strategy started at the three-way interaction term to then eliminate two-way interaction terms, and then eliminated main terms, starting with the larges *p*-value until the remaining terms all had a *p*-value of ≤0.20. Except for Table 2, the data are given as the means and their standard errors. The level of statistical significance was set to 0.05.

## 3. Results

Table 1 presents the mean age, anthropometrics, body mass, and countermovement jump for the participants.

### 3.1. Chronic Responses: Resting Baseline Levels

Table 2 lists the resting baseline levels of CTX, P1NP, and sclerostin before exercise. For CTX, there was no effect of group (*p* = 0.72), session (*p* = 0.52), or their interaction (*p* = 0.20). In contrast, P1NP showed a trend for session (*p* = 0.10), without any effect for group (*p* = 0.95) or the group × session interaction (*p* = 0.62). Sclerostin showed a significant effect for session (*p* = 0.039) but no effect for group (*p* = 0.14) or the group×session interaction (*p* = 0.29).

### 3.2. Acute Responses: Percentage Changes from Resting Baseline Levels

**Figure 2:**
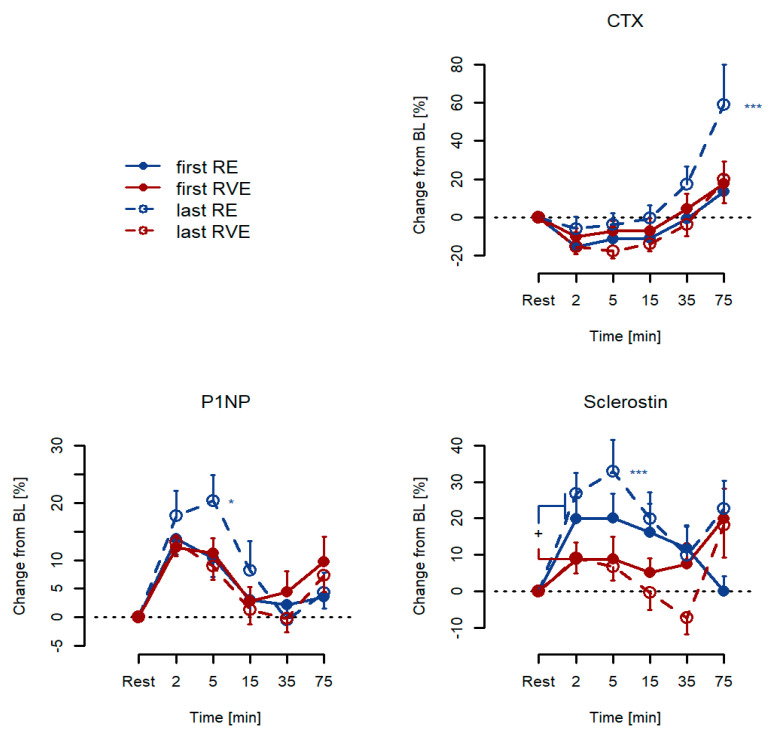
Acute responses to exercise training for serum CTX, P1NP, and sclerostin, given as percentage changes from resting baseline levels for both RE (*n* = 13) and RVE (*n* = 13) groups. * *p* < 0.05, *** *p* < 0.001.

The serum levels of CTX depicted a significant bi-phasic time course in response to exercise training (*p* < 0.001) that was comparable between groups (*p* = 0.66). This response consisted of reductions in the order of magnitude of −10 from 2 min to 15 min after exercise and with a larger increase at 75 min (all *p* < 0.001, except for 15 min where *p* = 0.004). In addition, there was a significant group × session interaction (*p* < 0.001), indicating that the acute exercise was exaggerated at the last session in RE only (Figure 2). The serum levels of P1NP also depicted a significant time response (*p* < 0.001) that was comparable between groups (*p* = 0.66). However, this response was mono-phasic and limited to 2, 5, and 15 min after exercise (*p* < 0.001, *p* < 0.001 and *p* = 0.026, respectively). No effect was observed at 35 min (*p* = 0.72) or 75 min (*p* = 0.12). Moreover, P1NP depicted a group × session interaction (*p* = 0.025) that indicated an exaggerated acute response at the last exercise training session in RE only. The sclerostin responses were somewhat more varied than those responses of CTX and P1NP. Thus, sclerostin responses were significant throughout the post-phase (*p* < 0.001 for +2, +5, and +15 min; *p* = 0.043 at +35 min; and *p* = 0.020 at 75 min), indicating an acute increase in the order of magnitude of 20% initially. For CTX and P1NP, there was a group×session interaction effect (*p* = 0.002) indicating an enhancement of the last session’s response in RE. Moreover, a group effect (*p* = 0.047) indicated that the response was generally greater in RVE than in RE, and a group*time effect (*p* = 0.003) implied that the acute responses differed at +2, +5, and +15 min post-exercise (all *p* < 0.05). Table 2 displays the changes in the serum levels of CTX, P1NP, and sclerostin among the study subjects categorized into RE or RVE groups and divided into the first and last training sessions, respectively.

In this regard, there were only marginal variations in the serum levels of CTX and P1NP for the first and last RE as well as RVE training sessions. Conversely, we found statistically significant differences in sclerostin responses between the first and last training sessions in the RE and RVE groups. Interestingly, at rest, the serum levels of sclerostin were higher in the RE group than in the RVE group (first and last training sessions). Furthermore, without the addition of vibration, the serum levels of sclerostin were diminished after a six-week training intervention.

Acute effects were tested at the first and last sessions of a 6-week training program. Significant changes from resting baseline levels were observed (time effect) for CTX, P1NP, and sclerostin (all *p* < 0.001). In addition, there was a session × group interaction effect observed for CTX and P1NP (*p* < 0.001 and *p* = 0.025, respectively, marked as *** and as *, respectively). For sclerostin, significant interaction terms were found for group × time and session × group (*p* = 0.003 and *p* = 0.002, respectively, the latter marked as ***: * *p* < 0.05, *p* < 0.01, and *** *p* < 0.001). The serum levels of CTX decreased by 15% within 1–15 min after training intervention for both the initial and final sessions. After RE, the levels returned to baseline and displayed an overshoot of 18% at 75 min after exercise.

## 4. Discussion

The primary aim of this study was to evaluate whether a six-week resistance training program with or without the addition of whole-body vibration would affect the metabolic markers of bone metabolism at the end of the intervention, at rest, and in response to the applied exercise protocol. The rationale for this was the observation that effective countermeasure exercise against bone loss was obviously not induced through suppression of bone resorption but rather through the stimulation of bone formation. Therefore, we were interested in whether increases in bone formation in an ambulatory training intervention study are as substantial as those in experimental bed rest studies [8]. Results from the present study clearly demonstrate that this was not the case. Therefore, it seems that RE training, with or without superimposed vibration, does not elicit any long-standing shifts in balance between bone resorption and bone formation in the ambulatory setting. Generally, bone modeling adapts the structure to loading and is the result of an uncoupled action of the bone formation and resorption processes, whereas bone remodeling is the structural result of a coupled resorption-reposition process [31]. Both of these processes are primarily responsible for the maintenance, construction, and reconstruction of the skeleton throughout life. In particular, bone remodeling enables the constant renewal of the skeleton. In this regard, bone resorption by osteoclasts and formation by osteoblasts are tightly coupled within a bone multicellular unit, and bone resorption always precedes bone formation. In support of this view, bone turnover has been considered to be essential for preserving the mechanical integrity of the skeleton and regulating calcium and phosphorus homeostasis [32]. It is therefore an intriguing hypothesis to speculate that countermeasure exercises that can successfully offset immobilization unfold their effectiveness through an action on the coupling of formation and resorption [33]. If so, then the question still remains as to why there is such a prominent increase in bone formation induced by the bed rest model.

The interpretation of these data is not trivial, because only inconclusive reports are available in the literature. Lombardi et al. [34] found elevated serum levels of sclerostin in male elite athletes performing weight-bearing activities compared with those performing non-weight-bearing activities. In contrast, 12 months of resistance training twice per week or 12 months of completing a jump protocol three times per week resulted in a 7% decrease in the serum levels of sclerostin in men [35]. Within this study, sclerostin responses were significantly elevated throughout the post-phase, indicating an increase in the order of 20% initially. Acute sclerostin responses were substantially greater in RE than RVE (*p* = 0.003), whereas no significant differences were noted in resting baseline levels of sclerostin from the beginning to the end of the six-week progressive training. Nevertheless, further results from this study provided evidence to demonstrate that training effects were actually seen in that six-week period. Specifically, positive effects of RE were found in gains in leg muscle size and function [25]. Furthermore, it appears that decreased serum levels of sclerostin are not related to further bone turnover markers (CTX and P1NP). In addition, as speculated by Pickering et al. [36], acute increase in the serum levels of sclerostin after exercise is likely due to the release of previously synthesized sclerostin from osteocytes into the blood flow, rather than to an increase in sclerostin gene expression due to the very short period of time [36]. However, in previous studies, the serum levels of sclerostin returned to baseline 1 h after exercise [37], whereas in the present study they remained elevated for up to 75 min after exercise. The changes might therefore be attributed to hemoconcentration. Unfortunately, we did not adjust for plasma volume changes in this study. According to the consistent levels of sclerostin post-exercise, a pre-release of sclerostin through osteocyte activity seems rather unlikely. The acute, post-exercise increase in circulating levels of sclerostin may be an indication of its endocrine-like effect on target tissues (i.e., adipose tissue), as previously proposed [38]. We have also obtained clear results with regard to our second study aim, namely, to assess the acute time course of bone metabolic markers to exercise, even though the results are quite complex. For CTX, for example, our study replicates previous findings of an immediate suppression in bone resorption that is more pronounced with RVE than with RE [22]. However, the initial decrease that we observed between +2 and +5 min after exercise of around 15% was reverted to its contrary +75 min later. Therefore, after RE the levels returned to baseline and displayed an overshoot of 18%. Such post-exercise increases in serum levels of CTX have been also observed 1–2 h after vigorous cycling, and that response seems to be mechanistically linked to calcium homeostasis [39]. For P1NP, the acute responses were limited to a time horizon of no longer than +35 min. Notably, P1NP increased by 12.9% (SD 9.1%) at 2 min after exercise, however, the effects that outlasted the +75 min observation interval were found for sclerostin only. An important question remains about the physiological nature of the observed transient effects. Are they caused by de novo synthesis of the compounds, is their physiological excretion from the bone modified, or are they a by-product of exercise-related fluid exchange between the bone’s extracellular and intravascular compartments? In any case, it emerges from the results of this study that acute responses do not predict the response to chronic exercise training.

This study has some limitations. First of all, this study has only involved young male participants, which may preclude generalization of results to female or older populations. Acute responses could potentially have been affected by exercise-related hemoconcentration. However, given that only four bouts with around 10 repetitions were performed, such effects must be expected to be rather small. Furthermore, we measured markers of bone turnover only in serum samples. The evaluation of bone biopsies in this case would probably allow a much more detailed understanding about bone response to mechanical stimulation. Next, bone marker responses were measured before and directly after (up to +75 min) the exercise session. Measuring marker responses at multiple time points would have provided more detailed information about bone marker response after exercise. Hence, this was related to the narrow window of subject availability. In order to achieve a more detailed understanding of metabolic bone marker regulation, a prolonged study period of eight weeks should be preferred in the planning of future studies.

## 5. Conclusions

The results of the present study did not demonstrate any sizable enhancement of bone turnover that could match the effects that have been repeatedly made in response to countermeasure exercise during bed rest. The question therefore stands regarding which mechanisms constitute the anti-catabolic effects that mediate countermeasure effectiveness in bed rest. In addition, both types of exercise systematically evoked acute, albeit small and transient, effects on bone remodeling in favor of resorption regarding the immediate post-exercise period. Moreover, these acute responses were found to be modified after the six-week training program. In general, therefore, caution needs to be exercised before extrapolating long-term effects from acute responses of bone metabolic markers. Overall, it is clear that there is a paucity of research on the biochemical response to high-impact exercise. It should also be noted that the results are highly variable, and only large and well controlled studies might help to further our understanding of bone metabolism in exercise and the role of circulating biochemical markers such as sclerostin in healthy subjects.

## Figures and Tables

**Table 1:** Baseline characteristics of the participating subjects.

Variable	RE Group (*n* = 13)	RVE Group (*n* = 13)	*p*-Value
Age, years	23.4 (±1.4)	24.3 (±3.3)	0.518
Body mass, kg	75.0 (±4.7)	74.7 (±6.9)	0.081
Height, m	1.79 (±0.05)	1.8 (±0.1)	0.309
BMI, kg/m^2^	23.4 (±1.4)	23.5 (±2.1)	0.113
CMJ height, cm	42.2 (±4.6)	41.7 (±2.2)	0.968

Values are presented as mean ± standard error of the mean. BMI = body mass index; CMJ = counter movement jump; RE = resistive exercise; RVE = resistive vibration exercise.

**Table 2:** Resting baseline levels of bone markers before the first and last exercise sessions for resistive exercise (RE) and resistive vibration exercise (RVE).

	First RE	First RVE	Last RE	Last RVE	*p*-Value [Group]	*p*-Value [Session]	*p*-Value [Group × Session]
CTX, ng/L	333.7 (±126.3)	332.6 (±192.9)	320.3 (±172.1)	368.1 (±144.0)	0.72	0.52	0.20
P1NP, µg/L	67.4 (±23.3)	68.2 (±24.3)	73.8 (±23.7)	71.0 (±18.9)	0.95	0.10	0.62
Sclerostin, ng/L	479.2 (±116.0)	400.0 (±132.8)	424.2 * (±104.2)	396.4 * (±147.5)	0.14	0.039 *	0.29

Data are given as means (±standard deviation). * *p* = 0.039 for the difference between first and last exercise; CTX = cross-linked C-terminal telopeptide of type I collagen; P1NP = pro-collagen type I amino terminal pro-peptide; RE = resistive exercise; RVE = resistive vibration exercise.
